# A viral video and pet lemurs on Twitter

**DOI:** 10.1371/journal.pone.0208577

**Published:** 2019-01-09

**Authors:** Tara A. Clarke, Kim E. Reuter, Marni LaFleur, Melissa S. Schaefer

**Affiliations:** 1 Department of Evolutionary Anthropology, Duke University, Durham, North Carolina, United States of America; 2 Pet Lemur Survey Initiative, housed by the University of Utah, Salt Lake City, Utah, United States of America; 3 Department of Anthropology, University of Utah, Salt Lake City, Utah, United States of America; 4 Lemur Love Inc., San Diego, California, United States of America; 5 Department of Anthropology, University of California San Diego, La Jolla, California, United States of America; 6 Anthropology Program, Salt Lake City Community College, Salt Lake City, Utah, United States of America; West Pomeranian University of Technology, POLAND

## Abstract

Content shared on social media platforms can impact public perceptions of wildlife. These perceptions, which are in part shaped by context (e.g. non-naturalistic setting, presence of a human), can influence people’s desires to interact with or acquire wild animals as pets. However, few studies have examined whether this holds true for wild animals featured in viral videos. This study reports on opportunistic data collected on Twitter before, during, and after a video that featured a habituated ring-tailed lemur (*Lemur catta*), called “Sefo”, in southern Madagascar went ‘viral’ (i.e. circulated rapidly on the internet). Our dataset of 13,953 tweets (from an 18.5-week time period in early 2016) referencing lemurs was collected using targeted keywords on the Twitonomy Service. We identified 613 individual tweets about people wanting a lemur as a pet. In addition, 744 tweets that were captured in our dataset linked to the Sefo viral video. We found that as the number of tweets about the viral video increased, so did the number of tweets where an individual wanted to have a lemur as a pet. Most tweets (91%) did not make reference to a specific species of lemur, but when they did, they often (82%) referenced ring-tailed lemurs (*L*. *catta*), ruffed lemurs (*Varecia* spp.), and mouse lemurs (*Microcebus* spp.). This study serves as a case study to consider how viral content can impact how wild animals are perceived. We close by noting that social media sites like Twitter, which are increasingly providing their users with news and information, should carefully consider how information about wild animals is shared on their platforms, as it may impact animal welfare.

## Introduction

### Background

The Internet has been gaining importance as an information source across the world [1]. By the end of 2016, almost half (47%) of the world’s population was using the Internet [2]. In the United States of America and in western Europe, many people are now getting their news via social media [3–5]. In mid-2017, for example, 67% of Americans reported getting at least some of their news from social media [6].

In the Western world, there is some evidence to suggest that science-related information is not shared or received by the viewer in the same way as non-science news on social media platforms. For example, a nationally-representative web-based survey of 4,024 U.S. adults administered by the Pew Research Center in June 2017 found that, in contrast to more general news, social media only played a small role in informing Americans about science [7]. This survey also found that, though most social media users saw science-related posts, only a quarter followed science social media accounts and 21% of users did not see any science-related posts on their social media at all [7]. In addition, 52% of social media users surveyed distrusted, rather than trusted (26% of users), social media posts that they saw about science [7]. For comparison, another survey–again administered by Pew Research Center, albeit in March 2017 of 4,151 U.S. adults–found that only 5% and 33% of social media users based in the United States have “a lot of trust” or “some trust”, in general, in the information they see on social media [8]. More broadly, there is evidence that social media websites can be an influential news source to the general public, especially when trust in traditional media sources decreases (in the United States [9], in Israel [10], across 11 countries [11]).

There are many examples of social media platforms being used in a way that results in positive conservation outcomes through the sharing of environmental or scientific information. For example, social media platforms have been used to increase support for integrated sustainability and conservation initiatives [12], raise funds for conservation (e.g., crowdfunding) [13], and serve as a place where people can voice their concern about the illegal extraction of threatened and endangered species from the wild [14]. The positive impacts of social media platform use for animal conservation efforts include the removal of videos of wild animals kept as pets that have been illegally taken from the wild from social media sites [14, 15] and its use to cost-effectively launch outreach-platforms aimed at bringing information about conservation programs in Madagascar, for example, to social media users in the United States and Europe [16, 17]. In another example, Barbary Macaque Conservation in the Rif launched a Facebook page to promote their research and raise awareness of the threats Barbary macaques (*Macaca sylvanus*) face in the wild, in particular being targeted for the pet trade [18]. This spurred their Facebook followers to report sightings of illegal pet macaques, resulting in four confiscations [18].

On the other hand, the information shared on social media sites can also have negative environmental outcomes, even if unintentional. This could be partly because only 16% to 35% of people pay attention to the source of news that they see on social media (in western European countries) [3]. The literature, which has focused primarily on more traditional media types (i.e. not social media) has shown that images presenting animals in a non-natural setting, especially where context is missing, can result in a range of misperceptions about wild animals [19–21]. For example, in Ross et al. 2011 [20], viewers who saw images of chimpanzees (*Pan troglodytes*) standing next to a human in a photograph, were 30% likely to want to own one as a pet and 35% more likely to assume the wild populations were not threatened. Likewise, Leighty et al. 2015 [22] found that photographs of capuchin monkeys (*Cebus sp*.), squirrel monkeys (*Saimiri sp*.), and ring-tailed lemurs (*Lemur catta*), in a non-natural setting in contact with a person increased their desirability as pets and increased the likelihood of viewers believing the animal was not endangered.

‘Viral’ information is one aspect of social media that remains understudied as it pertains to environmental information. Virality of content is driven in part by emotional experiences, as well as the strength of the emotion being felt [23], with pleasant emotions having a greater influence on sharing than unpleasant [24]. It is not rare for footage of animals, sometimes wild and often domestic, to be shared widely across the internet, occasionally ‘going viral’ [25]. The consequences of this viral information, though sometimes the subject of popular media stories [e.g. 25], have rarely been assessed in the peer-reviewed literature. One of the most well-known peer-reviewed examples is the 2009 viral YouTube video that showed a pet pygmy slow loris (*Nycticebus pygmaeus*) being ‘tickled’ by its owner [15, 26]. Although pygmy slow lorises are Vulnerable (VU) and protected via CITES Appendix I [27], up to one-quarter of commentators indicated that they wanted a slow loris as a pet [15]. The authors concluded that although few of the commentators were from slow loris range countries, the potential negative impacts of viral videos reinforcing people’s likelihood of wanting to acquire a slow loris as a pet could be high enough to warrant the inclusion of permanent warnings embedded in online videos of threatened species [15].

### Current study

**Fig 1:**
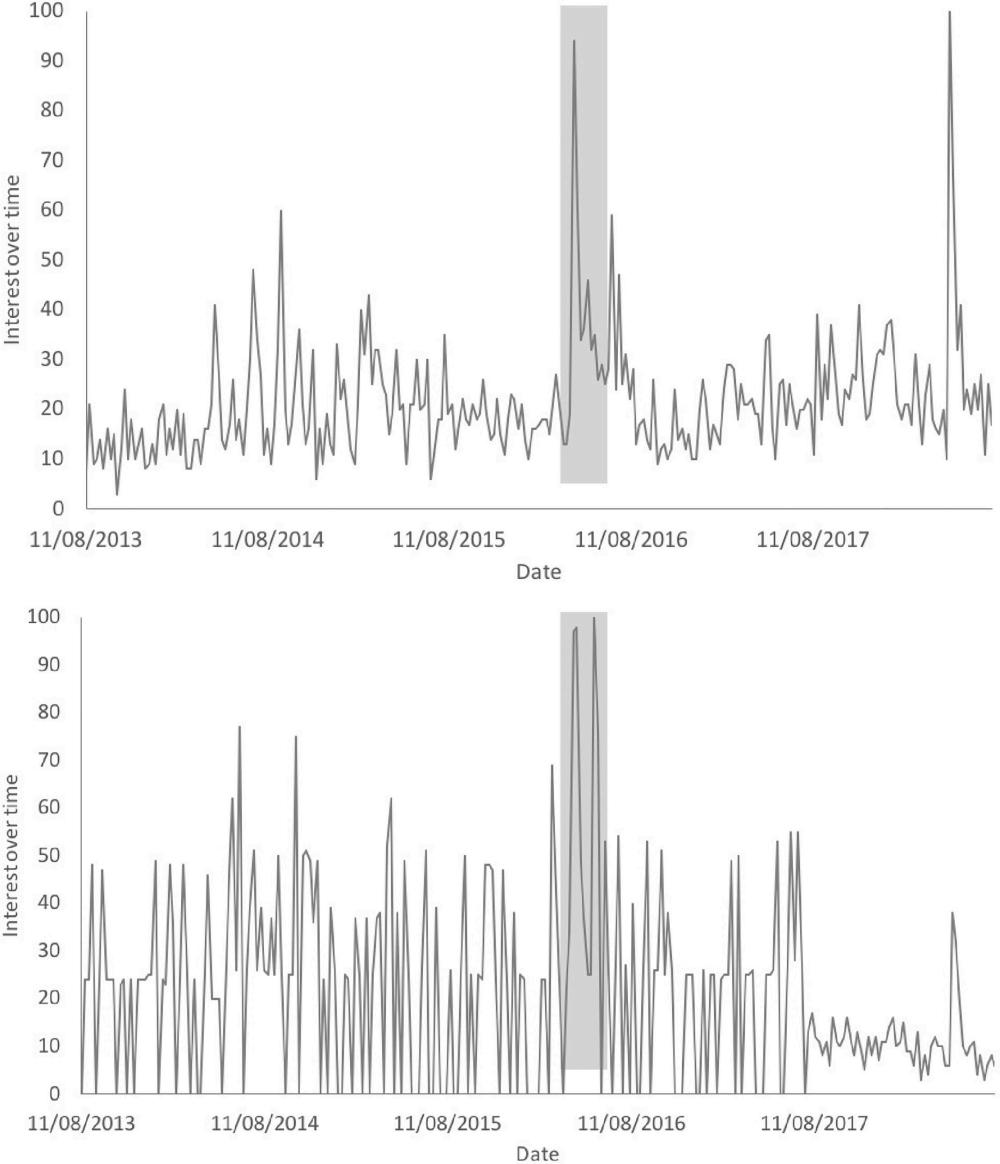
**Searches for the term ‘pet lemur’ on Google (A) and YouTube (B) from August 2013 to August 2018.** Information downloaded from the open-access Google Trends database. The y-axis, ‘interest over time’ is a metric derived by Google which is relative to the highest point on the chart (i.e. a value of 100 indicates peak popularity of the search term for the time period considered). The area shaded in grey shows the weeks during and just after the viral lemur video was shared on social media sites.

In April 2016, a ‘viral’ video made rounds on the Internet featuring two children in a rural area of Madagascar scratching a habituated ring-tailed lemur (*Lemur catta*), called “Sefo” (meaning ‘chief’), on the back (hereafter referred to as the ‘viral video’). The video showed the lemur patting its back (which was interpreted by most viewers to mean that the lemur was ‘asking’ for more back scratches from the children). This video was uploaded onto Facebook and YouTube in April 2016. The original Facebook post was viewed ~20 million times within a week of being published [28]. The video was shared widely on Facebook and on Twitter, as well as by several popular media platforms such as The Today Show (a morning talk show broadcast from New York City) [29], and online news articles aiming to educate the public on the context of the video. Viewership of the video decreased dramatically after the viral video ‘peaked’ (Fig 1) and the Facebook video has only accrued ~2 million more views in the almost 2.5 years since April 2016. The original YouTube video has since been removed, though it is still available on YouTube, having been uploaded to many YouTube accounts not associated with the original owner of the video.

The release of the video happened to coincide with ongoing data collection that the authors of this study had initiated, whereby we were pulling tweets about pet lemur ownership (using a series of keywords) from the Twitter Application Programming Interface (API) on a weekly basis. Twitter [30] is a social media platform with 328 million active users and is popular for news distribution in the United States of America (USA) [6], and the United Kingdom (21% of social media users use Twitter as a news source) [3]. Non-English speaking countries use Twitter as a news source infrequently compared to other social media sites [3]. Twitter is not the largest of the social media platforms, but in the USA, the number of users that receive news on Twitter has been increasing for several years (52% of users in 2013 to 59% in 2016 and 74% in 2017) [6]. In 2017, the portion of users who received news on Twitter in the USA was comparable to Facebook and Reddit but higher than YouTube, LinkedIn, and a range of other social media platforms [6].

In this study, we used the opportunity to examine whether a viral media image of a lemur in a non-natural setting (i.e. in a rural village in Madagascar, in contact with children) had an impact on how often people tweeted about wanting lemurs as pets. We present data collected before, during, and after the viral video was shared online.

## Materials and methods

### Lemurs

Lemurs are an ancient and diverse primate radiation found only on the island of Madagascar. They compose 20% of the world's primate species and 30% of family-level diversity, though they occupy a land mass that is just 1.3 to 2.9% of the other three land masses where extant nonhuman primates occur (Africa, Asia, Neotropics) [31]. Threats to lemur survival include habitat loss/degradation [32], bushmeat hunting [33, 34], and live-capture primarily for the within-country illegal pet trade [35]. More than 30 species of lemur have been reported as pets (defined as being wild captured and dependent upon humans for food) within Madagascar, with ring-tailed lemurs being particularly popular for the illegal, in-country pet trade [35].

### Collecting data from Twitter

In this study, we used data from Twitter [30] only. Initially, when we began collecting data (prior to the release of the ‘viral video’, which we could not have anticipated), our intent was to passively collect social media status updates about captive and pet lemurs. Our research questions had been: 1) how many people tweet about wanting lemurs as pets?; 2) what species of lemur do people often want to keep as a pet?; 3) how are people interacting with lemurs in person and in popular media?; and 4) do virtual or in-person interactions with lemurs seem to be linked with an online expression of interest to have a pet lemur?. It is within this framing, that we were opportunistically able to examine the impact of the viral video on our dataset.

We chose to collect data from Twitter, as opposed to other social media sites, for several reasons. First, and advised by two colleagues knowledgeable on the subject (see Acknowledgments), it was clear that Twitter would allow for the following (in contrast to other major social media sites): 1) Twitter allows for direct access to search user status updates and real-time information on trending keywords; 2) Twitter also provides access to a small amount of historic data, allowing us to pull information from the Application Programming Interface (API) once a week instead of more frequently; and 3) we could access Twitter data through third-party sites easily, and download the data into Excel for sorting and coding. In addition, the topics of discussion on Twitter were thematically overlapping with our research questions, hence our focus on this social media site as opposed to some other options. Because of these attributes, and the limitations on resources that would have allowed for data analysis from multiple platforms, we chose to focus our efforts on collecting data from Twitter. We acknowledge that data from other social media sites (particularly Facebook, YouTube, and Instagram) would have been useful to include in our study.

Data from Twitter were collected from January 1^st^ 2016 to May 7^th^ 2016 via the Twitonomy Service [36]. All ‘terms of service’ were complied with during data collection. Twitonomy is a service which, for a small fee, will pull data (such as tweets and information about the profiles of individuals publishing those tweets) from the API based on pre-selected keywords. Nineteen targeted English-language keywords (including plurals of all words where appropriate) were employed to capture tweets regarding captive and pet lemurs (Table 1). We focused on the English language for two reasons: 1) as a practical mechanism for keeping the dataset to a manageable size, and 2) because the study authors are all native English speakers.

**Table 1:** Keywords used to capture tweets regarding pet lemurs, number of tweets pulled from the Twitter API using each keyword, and number of tweets about people wanting to have a pet lemur.

Keywords used	Number of tweets (n = 13,953 tweets)	Number of tweets about wanting a pet lemur (n = 613 tweets from 582 people)
Zoboomafo*/Zaboomafo*	130	0
Want* lemur*	896	672
Touch* lemur*	50	0
Sell* lemur*	14	2
Purchase* lemur*	12	2
Pet* lemur*	1035	117
Own lemur*	5	2
Our lemur*	639	0
My lemur*	3934	89
Legal* lemur*	16	4
King* Julien*/ kingjulien	3178	2
Julien* lemur*	85	1
Illegal* lemur*	310	1
Have* lemur*	2423	53
Has* lemur*	960	5
Domestic* lemur*	7	0
Calm* lemur*	21	0
Buy* lemur*	227	46
Breeder* lemur*	3	1
Auction lemur	1	0
Aggressive lemur	7	0

Although only English-language keywords were utilized, tweets in other languages were occasionally captured in our search results. In these cases, good faith efforts were made to read any tweets in German and French (i.e., languages in which the authors had fluency). However, most of these German and French-language tweets were not directly relevant to the study and were excluded. Tweets in other languages were excluded from consideration. We did not use translation platforms like Google Translate as the accuracy in translation can be low [37, 38], especially when slang words are used or words are truncated (as we observed to be common in the English-language tweets), which is likely due to the character count limitation of tweets on Twitter.

We acknowledge that the use of pre-selected keywords (e.g. not using the keyword ‘monkey’ but focusing explicitly on lemurs at the outset) is a limitation of the paper. We chose these keywords through an iterative process whereby we identified keywords that pulled tweets from the Twitter API that directly responded to our initial research questions, but also did not result in an unmanageable dataset. It became clear during the keyword setting process, that individual tweets (and sometimes entire tweet conversations/chats) would need to be read by researchers (and that automated programs could not sort and categorize tweets for us) because of the nuance and context of many of the tweets. This process likely biased our dataset by excluding tweets where lemurs were referenced by the general public as monkeys or other animals. For example, several tweets were pulled from the Twitter API using our keywords (though we excluded these tweets from analysis) that showed people incorrectly using the word ‘lemur’ to refer to other types of animals (e.g., slow lorises, *Nycticebus* spp., for example; Nekaris et al. 2013 [15] noted that people commenting on online videos of slow lorises did sometimes refer to them as lemurs).

The resulting dataset contained almost 14,000 tweets (see Results) as well as some or all of the following information: date and time of the tweet; profile information of the author of the tweet; text of tweet; URL of tweet; and the number of interactions that others had with the tweet (e.g. number of re-tweets). Tweets were categorized as relevant if the tweet indicated: 1) that a person wanted to have a lemur as a pet and/or 2) linked to the viral video. We also recorded whether tweets involved or referenced human-lemur contact at a zoo and about privately-owned lemurs. These data and a brief analysis of tweets about human-lemur contact at zoos and about privately-owned lemurs can be found via S1 Table and S1 Fig.

Tweets were categorized as relevant via a three-step process, whereby the almost 14,000 unique tweets were manually read three separate times by the same researcher (KER) on different days (first when the data were downloaded each week from Twitonomy, a second time generally a few weeks later to compare against more recently downloaded tweets, and a third time at the end of the data collection period to compare against all tweets downloaded). This three-step process was instituted to limit the subjectivity of whether or not tweets were considered relevant to the study and the inclusion/exclusion of tweets was appropriate. If KER was not sure whether a tweet was relevant or not, the tweet was initially marked as ‘maybe relevant’ during the first read and then, in the second and third reads, the entire twitter conversation/chat within which the tweet was found (to look at any accompanying images or links that were included in the tweet) was reviewed in its entirety, to make a subjective determination as to whether the tweet was relevant or not. The additional step of examining contextual information usually served to exclude a tweet from consideration. We acknowledge that there is subjectivity as to whether or not tweets were included or excluded; the three-step process described above aimed to ensure that the same interpretation of tweets was used throughout the study. The full dataset is publicly available for download in the S1 Dataset.

In order to have consistent and reliable interpretation and classification of tweets, we established the following classification guidelines. First, tweets were classified as people ‘wanting a pet lemur’ if: 1) the tweet explicitly noted that a person wanted to own or have a lemur (whether or not that person indicated an ability to have the lemur; hereafter referred to as ‘wanting a pet lemur’); or 2) inquired about how to procure a lemur. Tweets where it was not clear what the person was tweeting about (e.g., when a person was using the word ‘lemur’ to refer to another person or a non-lemur animal) were excluded from the analysis, but only after the three-step process was followed (as described above), the entire twitter conversation/chat (and other materials linked to in the tweet) were considered, and when it was absolutely certain that the tweet was not relevant to this study. Notes were taken if the person referenced a specific species of lemur, referenced popular media, or posted a photo of a lemur.

In regard to including tweets about the viral video, we included only tweets that linked directly to the viral video. We did not consider tweets about lemurs that did not directly link to the video, even if the text of the tweet suggested that the author had watched the video.

### Statistical analyses

Statistical analyses were conducted in JMP software [39]. Averages presented in the results using n = 18 weeks as replicates, are excluding the incomplete 19^th^ week of data that was collected.

Kruskal-Wallis Rank Sums Tests were used to test whether the number of tweets about wanting a pet lemur differed by month (n = 4 months; with Wilcoxon Each Pair tests used as a post-hoc test).

A standard least squares regression was used to test whether the number of tweets about people wanting a pet lemur increased as references to the ‘viral video’ increased. In this analysis, the dependent variable was the proportion of people per week (n = 18 weeks) who tweeted a link to the viral video (excluding those who—in the same tweet—stated that they wanted a lemur). The independent variable was the proportion of people who stated that they wanted a lemur per week (n = 18 weeks; excluding those who—in the same tweet—linked to the viral video). The number of ‘viral video’ tweets was natural log-transformed prior to analysis to meet assumptions of normality.

## Results

### Parameters of the dataset

The dataset contained 13,953 tweets, collected during an 18.5-week period (January 1, 2016 through May 7, 2016), of which 2,294 (16%) were deemed broadly relevant in that pet or captive lemurs were the topic of the tweets (full dataset publicly available for download in S1 Dataset). Keywords primarily solicited results in the English language.

A total of 613 (4%) tweets expressed someone ‘wanting a pet lemur’ and 774 (6%) tweets linked to the ‘viral video’ (see Methods). It should be noted that these are not mutually exclusive, and 18 individuals who referenced the viral video (out of n = 765 tweets about the viral video) also stated that they wanted a lemur as a pet.

A further 148 (1%) tweets described human-lemur contact at a zoo and 359 (3%) tweets referenced a privately-owned pet lemur (a lemur not kept at a zoo). The rate that tweets were published about human-lemur contact at zoos (i.e. tweets published per week) did not change during the duration of the study (see S1 Fig.). Tweets about both human-lemur contact at zoos and privately-owned lemurs did not increase as tweets referencing the viral video increased (see S1 Fig.). A large number of tweets in our dataset were included as a result of the keywords targeting references to ‘King Julien’ and to ‘Zaboomafoo’ (n = 3,263 for ‘King Julien’ keywords; n = 130 for the ‘Zabomafoo’ keywords). However, they were rarely mentioned in tweets where people stated that they wanted to own pet lemur or where people reported having seen or otherwise interacted with a lemur in captivity (<1% of tweets, n = 28 tweets, S1 Fig.). These data are not discussed further in this paper, but a brief analysis is provided via S1 Appendix and S2 Appendix.

### Wanting lemurs as pets

**Fig 2:**
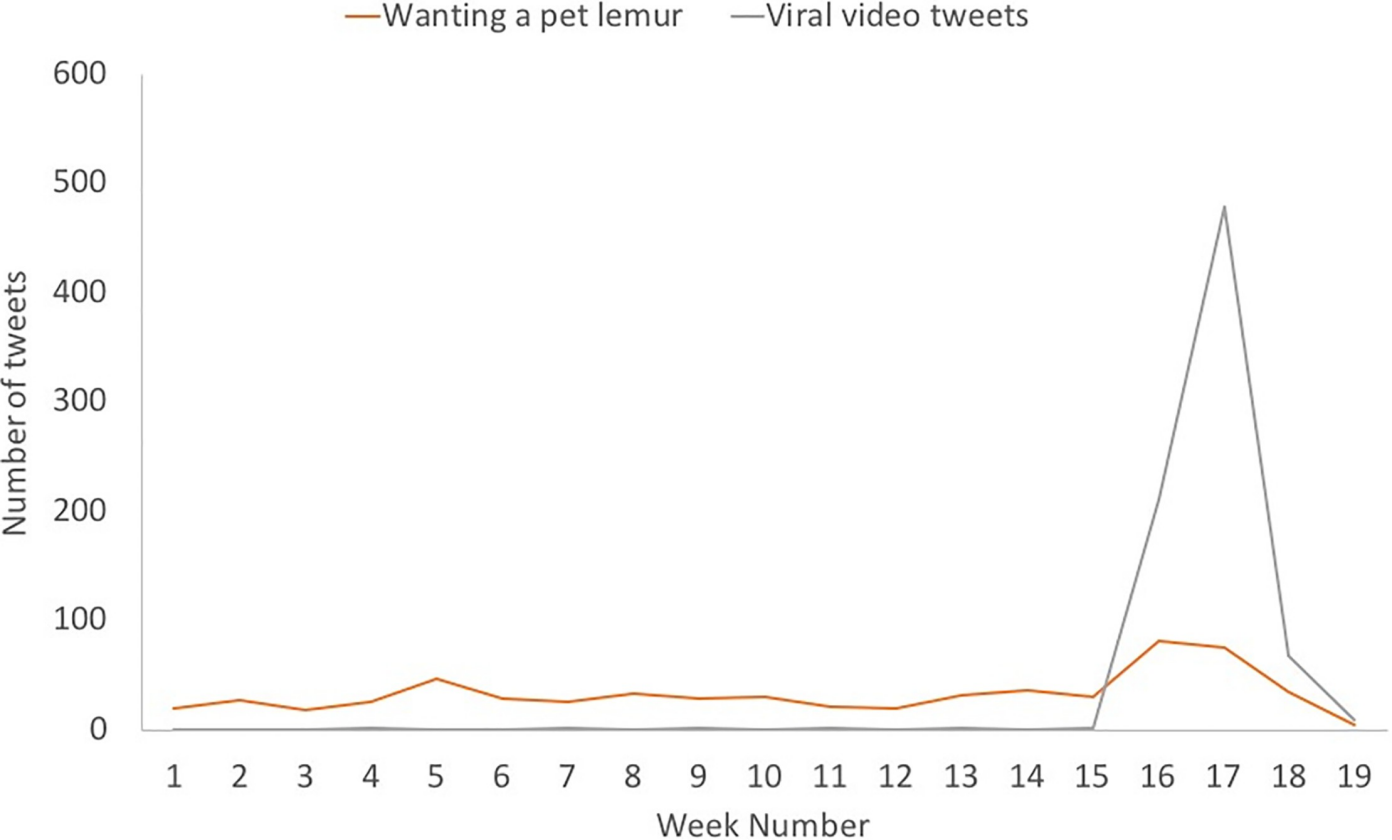
Number of tweets indicating someone wanting to own a pet lemur and number of tweets linking to the ‘viral video’.

**Fig 3:**
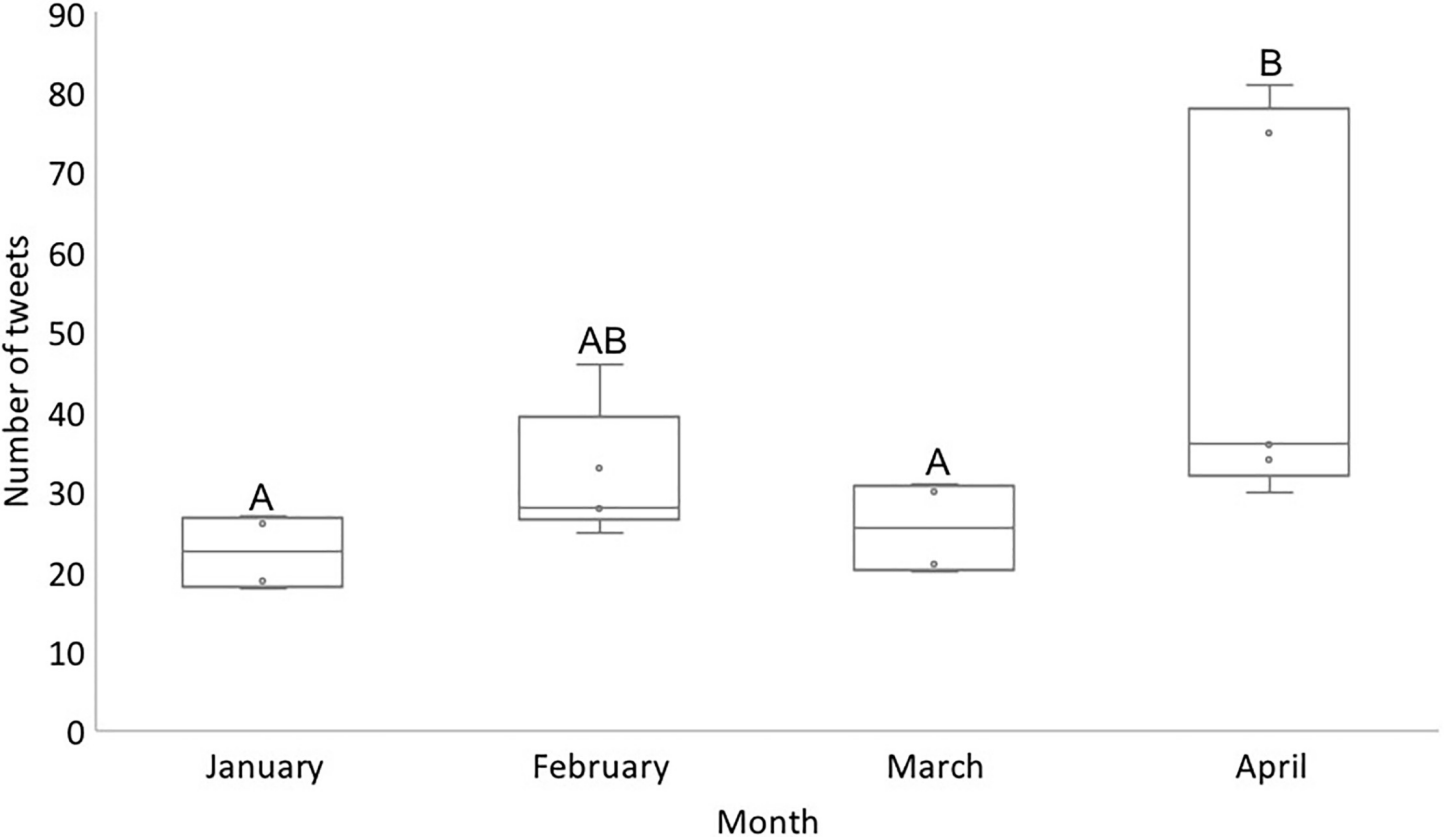
Number of tweets published by month indicating someone wanting to own a pet lemur (with weeks as replicates). Letters indicate significant differences. The boxes highlight the quartiles while the whiskers indicate the variability outside the outer quartiles.

A total of 613 (4%) tweets from 582 Twitter users over 18.5 weeks were about a person wanting a lemur as a pet (34 ± 17 tweets per week or 5% ± 3% of tweets in our total dataset per week, mean ± st. dev., n = 18 weeks; Fig 2). The number of tweets published about wanting lemurs as a pet differed by month (Kruskal-Wallis Rank Sums Test, Chi-square = 10.1020, DF = 3, P = 0.0177, weeks as replicates within n = 4 months; Fig 3). The number of tweets published about someone wanting a pet lemur was higher in April than in January (Wilcoxon Each Pair Test, Z = 2.237, P = 0.0200) and March (Wilcoxon Each Pair Test, Z = 1.968, P = 0.0491), but not different than in February (Wilcoxon Each Pair Test, Z = 1.845, P = 0.0651). The number of tweets about someone wanting a pet lemur were fairly low (≤ 46/week) throughout the study period except for in the two weeks coinciding with the release of the viral video (Fig 2). Note that we did not find any evidence that people were actively buying or selling lemurs using Twitter.

### The viral video

Many tweets were captured in our dataset that linked to the ‘viral video’ (see Methods, n = 744 tweets). The tweets that linked to the viral video did not contain references to other lemurs that people may have seen or interacted with in captivity. Most of these tweets were published in April 2016 (27%, n = 210 in week 16 of our dataset; 62%, n = 479 in week 17; 9%, n = 68 in week 18; Fig 2). Only 19 tweets were published on Twitter linking to this video outside of weeks 16, 17, and 18 (Fig 2). The number of tweets about someone wanting to own a pet lemur increased with the number of tweets published linking to the viral video (Standard Least Squares Regression: F-Ratio = 3.9673, DF = 3, P = 0.0328, R^2^ = 0.676094).

### Lemur species referenced in tweets

Of the 613 individuals that tweeted about wanting a pet lemur as a pet, 91% (n = 555) did not provide any indication as to what species they were referencing. In some cases, however, the tweets explicitly named a species or was accompanied by a photograph. The following species were mentioned or featured in photographs: *Lemur catta* (n = 47), *Varecia rubra* (n = 4), *Microcebus sp*. (n = 3), *Eulemur collaris* (n = 1), *Propithecus sp*. (n = 1), and *V*. *variegata* (n = 1). *Lemur catta* and *V*. *rubra* were two of the three species that people most commonly tweeted about coming into contact with (see S2 Table). In addition, *L*. *catta* was the species of lemur featured in the ‘viral video’.

## Discussion

In this study, we found that a small but consistent number of people are tweeting about wanting a lemur as a pet (4% of our dataset over 18.5 weeks), even as we found no evidence of buying or selling (legally or illegally) of lemurs using the Twitter platform. Though only 9% of people referred to specific species of lemurs in their tweets, when they did, they often (82% of the time) referenced ring-tailed lemurs (*L*. *catta*). We found that the number of tweets about someone wanting to own a pet lemur increased with the number of tweets published linking to the viral video. It is clear that the direct threat to wild lemur species from the viral video is very low. We consider, however, the indirect implications of this video as another instance in which anthropomorphized media of lemurs could incorrectly influence public perception about the threatened status of lemurs in Madagascar.

### Wanting a lemur as a pet

The number of people who ordinarily tweet, in English, about wanting a lemur as a pet is small, but our data indicate that the number of tweets where someone wanted a pet lemur increased as the number of tweets linking to the viral video increased. This increase was not observed when looking at other types of lemur interactions that people tweeted about (e.g. lemurs in zoos or privately held lemurs, see S1 Fig.). This is interesting information, even if the absolute number of people tweeting about wanting a pet lemur is low, because it provides a context for how viral videos can reinforce interest in having wild animals as pets (a concept discussed by [15]). Experimental studies have shown that when people are shown a photograph of primates in non-natural settings together with people (compared to control photographs of primates in natural settings), there is an increase in the likelihood of viewers wanting to own a primate as a pet [20, 22]. Our study suggests that this pattern is true also for viral videos.

### Ring-tailed lemurs in popular culture

It is not surprising that ring-tailed lemurs were the most popular species mentioned in our tweet dataset, even beyond the fact that it was a ring-tailed lemur that was featured in the viral video. Ring-tailed lemurs are the most commonly kept primate in captivity in the world, and it was estimated that over 2,500 were being kept in zoos in 2009 [40]. Almost one-third of Americans (30%) have encountered science information in the past year at a zoo or aquarium [7], and our dataset included many people tweeting about the ring-tailed lemurs they were able to interact with at these zoos (see S1 Fig.). In addition, ring-tailed lemurs have become more visible to the general public and have been propelled into popular culture. For example, DreamWork’s Madagascar released a feature-length animated film, *Madagascar*, in 2005, which starred ‘King Julien’ an anthropomorphized ring-tailed lemur. The franchise now has a total of three feature length films and a television series spin-off entitled *All Hail King Julien*. To date, the franchise has grossed over 564.1 million USD (worldwide unadjusted revenue) [41].

### Conservation implications

It is clear that the direct threat to lemurs in the wild from the sentiments expressed on Twitter, at least as captured in this study, is very low (as lemurs cannot be extracted from the wild and exported out of Madagascar for the pet trade [42]). Pet lemurs in the United States of America and the United Kingdom (where the majority of our tweets were likely from), are sourced from captive-bred populations that were taken out of Madagascar many decades ago. This is in contrast to the Nekaris et al. 2013 [15] study where captive reproductive success for slow lorises is low enough that most lorises in pet shops do not come from accredited breeding facilities and are instead taken illegally from the wild.

However, this study serves to make the case that viral video content (which in this case reached at least 20 million people in a matter of weeks) could reinforce misperceptions about wild animals and increase the number of people in a population who want to have one as a pet. This is not a novel concept, having been examined–with more or less robust analyses–for the movie industry across many studies. For example, there were increases in Dalmation registrations seven years after the release of Disney’s *101 Dalmations* film, which may have been attributable to the film (though not conclusively) [43], as well as studies, on the global trade of green iguanas (*Iguana iguana*) in the three years after the release of the first *Jurassic Park* film [44] and the owl pet trade in Indonesia following the *Harry Potter* films [45]. However, more in-depth analyses have not found a link between the movie industry and an increase in the ownership of wild animals as pets, including one study on clown fish sales following the release of Disney’s *Finding Nemo* film in 2003 [46] and another on the owl pet trade in the UK following the *Harry Potter* films [47].

We echo some of the discussion points highlighted in Nekaris et al. 2013 [15], whereby the authors call for better regulations of media sharing websites such that content showing wild animals can be flagged, presented to the viewer with a disclaimer, or removed. Our research raises the question of the role and responsibility of Twitter as a platform where potentially harmful information about wild animals can be easily shared to potentially millions of users in a very short period of time. Twitter policies do not address animal welfare issues specifically, but broadly state that, “You may not use our service for any unlawful purposes or in furtherance of illegal activities. By using Twitter, you agree to comply with all applicable laws governing your online conduct and content” [48]. In mid-2017, Twitter appeared to be piloting a mechanism whereby users could flag offensive content [49], but this feature has not yet been instituted.

Other large internet companies have instituted relatively simple policies, with the aim of benefiting wild animals–either in captivity or in the wild. In 2016, TripAdvisor (an online travel site with 455 million unique visitors per month and listings for 7.5 million hotels, restaurants, and attractions) [50], banned ticket sales to attractions that allowed human contact with wild animals because of animal welfare concerns [51]. In 2009, ebay banned the sale of illegal elephant ivory on its site [52], though recent news reports suggest that this policy is not very effective [53].

We also encourage well-meaning actors, like conservationists, primatologists, and/or non-governmental organizations (NGOs) to take a cautious and thoughtful approach with the imaging (photos, videos) and conservation messaging that they develop and share online. As there are, and have been, instances where this was not the case. For example, in 2016, the Madagascar Office of National Tourism (ONTM) launched a campaign on Facebook and Twitter to promote travel to Madagascar by asking tourists to post photos of themselves with captive lemurs [54]. These photos were posted online without much context, and some showed illegally captive lemurs. Following outreach to ONTM, the photos were removed, before they had a chance to be shared too widely on the internet. We show here, the unintentional sharing of images/video featuring lemurs in non-natural settings and close to humans, can have indirect impacts on how English-speaking people perceive these wild animals. This may also be the case in Madagascar, where there is already published anecdotal evidence that ‘selfies’ posted with pet lemurs (often on Facebook) can show wealth or are considered ‘cool’ [55]. Therefore, any conservationists, primatologists, and/or non-governmental organizations (NGOs) should be aware of this context when they put information about lemurs on social media, as there is the risk that the information is misperceived both by English and non-English speaking audiences.

## Supporting information

S1 Appendix Additional methods and analyses. (DOCX)

S2 Appendix Additional results. (DOCX)

S1 Table Keywords used to capture tweets regarding pet lemurs, number of tweets using each keyword, number of tweets people expressing desire for a pet lemur, and number people tweeting about human-lemur contact (zoos and privately owned). (DOCX)

S2 Table Species of lemur with which people on Twitter had interacted and IUCN red list status. (DOCX)

S1 Fig Number of tweets indicating someone wanting to own a pet lemur, number of tweets linking to the ‘viral video’, number of tweets about someone seeing a privately-owned pet lemur, and number of tweets about human-lemur interactions at zoos. (DOCX)

S1 Dataset Dataset for PLOS ONE. (XLSX)
