# Supplementary material for: A viral video and pet lemurs on Twitter
Source: PLoS One. 2019 Jan 9;14(1):e0208577. doi: 10.1371/journal.pone.0208577 (PMC6326470; doi:10.1371/journal.pone.0208577)
Supplement: S1 Appendix — (DOCX) [file pone.0208577.s001.docx]

**Supporting Information**

**S1 Appendix: Additional methods and analyses**

In addition, to the analyses presented in the main body of the manuscript, we undertook a series of analyses about other aspects of our dataset that we provide here as additional context.

Please refer to the methods in the main body of the manuscript as these are just additional notes relevant to the analyses in this document.

*Tweets regarding ‘human-lemur contact’ at zoos:* This included any tweets where it was apparent that an individual had been able to touch or get within 1 meter of a lemur without a separation barrier (e.g., such as glass). A ‘zoo’ was defined as a legal captive facility where people could see lemurs in captivity (e.g., indoor/outdoor enclosures, free-ranging) during publicly stated, regular hours. This included facilities with free-ranging exhibits in which patrons could walk through the exhibit, as well as exhibits in which patrons were separated from the lemurs via enclosure, moat, etc. Tweets were excluded when the proximity of the human to the lemur could not be clearly established. This excluded individuals who were zookeepers that were tweeting about their interactions with lemurs that they were paid to take care of as part of their normal professional duties. Tweets where it was not clear whether the photo or tweet was about a ‘zoo’ (as per our definition) were classified as tweets regarding lemurs from non-zoo facilities (see, below). Notes were taken if the person referenced a specific species of lemur, used hashtags in the tweet, referenced popular media, or posted a photo of a lemur.

*Tweets regarding lemurs from non-zoo facilities:* This included: 1) people tweeting about touching or meeting lemurs in areas that were not zoos; 2) people tweeting about their own pet lemurs; and 3) people tweeting about other people’s pet lemurs (both lemurs they had personally seen or lemurs that they had not seen in person). Tweets were excluded if: 1) it was not clear what the person was tweeting about (e.g., when a person was using the word ‘lemur’ to refer to another person or a non-lemur animal); and 2) people mistakenly used the word ‘lemur’ to refer to a non-lemur animal (e.g., a slow loris). Notes were taken if the person referenced a specific species of lemur, used hashtags in the tweet, referenced popular media, or posted a photo of a lemur.

As noted in the main body of the text (Table 1), our search keywords specifically included reference to two different popular anthropomorphized lemurs: 1) ‘King Julien’ (a fictional, cartoon ring-tailed lemur in DreamWork’s ongoing Madagascar franchise) and; 2) ‘Zaboomafoo’ (a live-action children’s show which stopped being filmed in 2001 but is still in syndication, featuring a coquerel’s sifaka, *Propithecus coquereli*) (Table S1).

*Statistical Analysis:* Statistical analyses were conducted in JMP software [27]. Pearson Chi-Squared Tests were used to test: 1) whether the proportion of tweets referencing human-lemur interactions in zoos changed over time; and 2) whether the proportion of tweets referencing private pet lemur interactions changed over time.

A liner regression was used to test whether the number of tweets about people wanting a pet lemur increased as the number of tweets about interactions with lemurs in zoos and privately-owned lemurs increased. In these analyses, tweets were excluded when they could be categorized as being both about wanting a pet lemur *and* about a lemur interaction such as described above.
