# Supplementary material for: A viral video and pet lemurs on Twitter
Source: PLoS One. 2019 Jan 9;14(1):e0208577. doi: 10.1371/journal.pone.0208577 (PMC6326470; doi:10.1371/journal.pone.0208577)
Supplement: S2 Appendix — (DOCX) [file pone.0208577.s002.docx]

**Supporting Information**

**S2 Appendix: Additional results**

A total of 148 (1%) tweets described human-lemur contact at a zoo. These were posted to twitter at a rate of 8.11 ± 3.52 per week (mean ± st. dev, n = 18 weeks; Fig. S1). Individuals on Twitter named 42 different zoos in Australia (n = 2), Canada (n = 1), Hungary (n = 1), Ireland (n = 1), New Zealand (n = 1), Portugal (n = 1), Singapore (n = 1), South Africa (n = 1), Sweden (n = 1), United Kingdom (n = 18), and the United States (n = 12) where they had ‘human-lemur contact’ (see Methods for definition).

Over the 18-week dataset (excluding the last half-week of data), there was no change in the proportion of tweets per week that referenced human-lemur interactions in zoos (Pearson Chi-square Ratio, Chi-square = 25.327, DF = 17, P = 0.0876; Fig 1). Four individuals indicated they wanted a lemur as a pet in the same tweet where they tweeted about a human-lemur interaction in a zoo. The number of tweets about interacting with lemurs at zoos (human-lemur interaction) did not change with the number of tweets about wanting a pet lemur (Linear regression: F-Ratio = 0.0465, DF = 17, P = 0.8320; n = 18 weeks are replicates, four tweets excluded where the same tweet had content about visiting a zoo and about wanting a lemur as a pet).

A total of 359 (3%) tweets referenced privately-owned pet lemurs (a lemur not kept at a zoo). These were posted to twitter at a rate of 19.61 ± 7.96 per week (mean ± st. dev, n = 18 weeks; Fig. S1). There was a change in the number of tweets per week posted to Twitter regarding private pet lemur interactions (Pearson Chi-Square Ratio, Chi-square = 80.874, DF = 17, P < 0.0001; Fig 1). The number of tweets related to seeing lemurs as private pets did not change with a number of people tweeting about wanting a lemur as a pet (Liner Regression: F-Ratio = 0.0022, DF = 17, P = 0.9634).

Very few people (<1%) tweeting about anthropomorphized lemurs (e.g. ‘King Julien’ and ‘Zaboomafoo’) expressed a desire to have a lemur as a pet.

*Species with which individuals reportedly interacted:* A variety of nocturnal, diurnal, and cathermeral lemur species, ranging from Near Threatened to Critically Endangered, were named in our dataset (see Table S2). The majority of human-lemur contact at zoos occurred with ring-tailed lemurs (52%). Likewise, the most prevalent lemur species being kept as a privately owned pet were ring-tailed lemurs (34%).
