# Supplementary material for: A viral video and pet lemurs on Twitter
Source: PLoS One. 2019 Jan 9;14(1):e0208577. doi: 10.1371/journal.pone.0208577 (PMC6326470; doi:10.1371/journal.pone.0208577)
Supplement: S1 Table — (DOCX) [file pone.0208577.s003.docx]

**S1 Table. Keywords used to capture tweets regarding pet lemurs, number of tweets using each keyword, number of tweets people expressing desire for a pet lemur, and number people tweeting about human-lemur contact (zoos and privately owned).**

| **Keywords used** | **Any kind of lemur contact, inclusive of human-lemur contact at zoos and exposure to privately owned pet lemurs**  **(n = 599 tweets)** |
| --- | --- |
| Zoboomafoo*/Zaboomafo* | 20 |
| Zoboomafo | 2 |
| Want*s lemur*s | 240 |
| Wants lemur | 2 |
| Want lemur | 3 |
| Want lemur | 19 |
| Touch* lemur*s | 72 |
| Touch lemur | 5 |
| Sell* lemur*s | 1 |
| Sell lemur | 0 |
| Purchase* lemur*s | 0 |
| Purchase lemur | 0 |
| Pets* lemur*s | 1046 |
| Pets lemur | 0 |
| Pet lemurs | 12 |
| Pet lemur | 86 |
| Own lemur* | 0 |
| Our* lemur*s | 427 |
| Our lemur | 20 |
| My* lemur*s | 26572 |
| My lemur | 193 |
| Legal* lemur*s | 10 |
| Legal lemur | 1 |
| Kking* Jjulien*/ kingjulien | 12 |
| King Julien | 10 |
| Julien* lemur*s | 20 |
| Julien lemur | 2 |
| Illegal* lemur*s | 21 |
| Illegal lemur | 1 |
| Have* lemur*s | 9342 |
| Have lemur | 51 |
| Has* lemur*s | 3512 |
| Has lemur | 23 |
| Domestic* lemur*s | 0 |
| Domestic lemur | 0 |
| Calm* lemur*s | 10 |
| Calm lemur | 1 |
| Buy* lemur*s | 20 |
| Buy lemur | 2 |
| Breeder* lemur*s | 10 |
| Breeder lemur | 1 |
| Auction lemur | 0 |
| Aggressive lemur | 0 |

One keyword ‘handraised lemur*’/‘hand-raised lemur*’Other keywords were also used but did not result in any search results. Some tweets were pulled from more than one keyword; since duplicate tweets were deleted, the number of tweets pulled using different keywords may differ from what is presented in this table.
