# Supplementary material for: A viral video and pet lemurs on Twitter
Source: PLoS One. 2019 Jan 9;14(1):e0208577. doi: 10.1371/journal.pone.0208577 (PMC6326470; doi:10.1371/journal.pone.0208577)
Supplement: S2 Table — (DOCX) [file pone.0208577.s004.docx]

**S2 Table. Species of lemur with which people on Twitter had interacted and IUCN Red List status.**

| **Species** | **Conservation Status** | **Human-lemur contact at zoo**  **(n = 103 out of 148 tweets)** | **Privately owned pet lemur**  **(n = 157 out of 358 tweets)** | **Any kind of lemur contact, inclusive of human-lemur contact at zoos and exposure to privately owned pet lemurs**  **(n = 267 out of 577 tweets)** |
| --- | --- | --- | --- | --- |
| *Eulemur sp.* | - | 2 | 6 | 9 |
| *Eulemur fulvus* | Near Threatened (NT) | - | 2 | 2 |
| *Eulemur macaco* | Vulnerable (VU) | - | 2 | 2 |
| *Hapalemur sp.* | - | 1 | - | 1 |
| *Lemur catta* | Endangered (EN) | 77 | 121 | 198 |
| *Lepilemur sp.* | - | - | 1 | 1 |
| *Microcebus sp.* | - | - | 1 | 1 |
| *Propithecus sp.* | - | - | 2 | 2 |
| *Propithecus coquereli* | Endangered (EN) | - | 1 | 1 |
| *Varecia rubra* | Critically Endangered (CRE) | 14 | 4 | 20 |
| *Varecia variegata* | Critically Endangered (CRE) | 17 | 20 | 41 |

Species designations are based on photographs posted with tweets.
