# Supplementary material for: A viral video and pet lemurs on Twitter
Source: PLoS One. 2019 Jan 9;14(1):e0208577. doi: 10.1371/journal.pone.0208577 (PMC6326470; doi:10.1371/journal.pone.0208577)
Supplement: S1 Fig — (DOCX) [file pone.0208577.s005.docx]

**S1 Fig. Number of tweets indicating someone wanting to own a pet lemur, number of tweets linking to the ‘viral video’, number of tweets about someone seeing a privately-owned pet lemur, and number of tweets about human-lemur interactions at zoos.**
